# Characterization of the Transcriptional Complexity of the Receptive and Pre-receptive Endometria of Dairy Goats

**DOI:** 10.1038/srep14244

**Published:** 2015-09-16

**Authors:** Lei Zhang, Xiao-Peng An, Xiao-Rui Liu, Ming-Zhe Fu, Peng Han, Jia-Yin Peng, Jing-Xing Hou, Zhan-Qin Zhou, Bin-Yun Cao, Yu-Xuan Song

**Affiliations:** 1 College of Animal Science and Technology, Northwest A&F University, Yangling, Shaanxi 712100, P.R. China

## Abstract

Endometrium receptivity is essential for successful embryo implantation in mammals. However, the lack of genetic information remains an obstacle to understanding the mechanisms underlying the development of a receptive endometrium from the pre-receptive phase in dairy goats. In this study, more than 4 billion high-quality reads were generated and *de novo* assembled into 102,441 unigenes; these unigenes were annotated using published databases. A total of 3,255 unigenes that were differentially expressed (DEGs) between the PE and RE were discovered in this study (*P*-values < 0.05). In addition, 76,729–77,102 putative SNPs and 12,837 SSRs were discovered in this study. Bioinformatics analysis of the DEGs revealed a number of biological processes and pathways that are potentially involved in the establishment of the RE, notably including the GO terms proteolysis, apoptosis, and cell adhesion and the KEGG pathways Cell cycle and extracellular matrix (ECM)-receptor interaction. We speculated that *ADCY8, VCAN, SPOCK1, THBS1*, and *THBS2* may play important roles in the development of endometrial receptivity. The *de novo* assembly provided a good starting point and will serve as a valuable resource for further investigations into endometrium receptivity in dairy goats and future studies on the genomes of goats and other related mammals.

**E**mbryo implantation is a complex initial step in the establishment of successful pregnancy in mammals^1^ and consists of apposition, adhesion and invasion^2^. The synchronized differentiation of the receptive endometrium (RE) from the pre-receptive endometrium (PE) is essential for embryo implantation^3^. The development of endometrial receptivity is known as the “window of implantation” because it is a spatially and temporally restricted stage^4^. During this period, the endometrium undergoes pronounced structural and functional changes induced by the ovarian steroids oestrogen and progesterone, which prepare it to be receptive to adhesion and subsequent invasion by the embryo^5^,^6^. Studies have shown that infertility is partly caused by dysfunction of the receptive endometrium^7^. Furthermore, impaired uterine receptivity is one of the major reasons for the failure of embryo transplantation in humans and other mammals during assisted reproduction with good-quality embryos^8^,^9^.

The development of novel, high-throughput sequencing techniques has provided new strategies that can be used to analyse the functional complexity of the transcriptome^10^. There are three high throughput sequencing methods that can be used for transcriptomic studies, including the classical 454 pyro-sequencing method and the low-cost Solexa sequencing method; these methods have been employed frequently over the past few years^11^, but now Illumina sequencing has grabbed that first spot. The RNA sequencing (RNA-Seq) approach, which was developed to help analyse global gene expression, is an efficient method to map and quantify the transcriptome^12^. The holistic view of the transcriptome and its organization provided by the RNA-Seq method has revealed many novel transcribed regions, splice isoforms, and single nucleotide polymorphisms (SNPs), and has allowed the refinement of gene structures^13^,^14^,^15^,^16^,^17^. Finally, RNA-Seq generates absolute rather than relative gene expression measurements, thereby providing greater insight and accuracy than do microarrays^18^,^19^.

Notably, recent studies have reported that the attainment of endometrial receptivity is a complex process involving numerous molecular mediators^4^. Molecular studies have extensively investigated the possible genes involved in the establishment of the receptive endometrium^20^, such as hormones^21^,^22^, cytokines^23^, and growth factors^24^. Nevertheless, the molecular mechanisms involved in the development of the endometrium from the pre-receptive state to the receptive state remain largely unknown, and the complexity of the goat transcriptome has not yet been fully elucidated. Drawing on the experience of previous studies, in this study we adopted the Illumina RNA-Seq approach to obtain a larger and more reliable transcriptomic dataset^25^ from the PE (gestational day 5) and RE (gestational day 15) in dairy goats. Then, we constructed a comprehensive analysis of the endometrial transcriptional profiles at the global level to compare the genes expressed in the PE and RE and further explore DEGs, single nucleotide polymorphisms (SNP) and simple sequence repeat (SSR) using Gene Ontology (GO) and Kyoto Encyclopedia of Genes (KEGG) for DEGs. Therefore, the results of our present study may provide essential information in support of further research on the development of endometrial receptivity in dairy goats. Furthermore, our transcriptomic study will provided good reference data for gene expression profiling of goats.

## Results

### Sequencing Results

#### Summary of sequencing

This study used RNA-Seq to compare the transcriptomic landscapes of the endometrium from the PE (gestational day 5) and RE (gestational day 15) phases of 20 healthy, 24-month-old multiparous dairy goats. Total RNA from the receptive and pre-receptive endometria were used to construct RNA libraries for Illumina sequencing. Reads with adapters and low quality reads were removed prior to assembly. In total, we acquired 46,514,662 and 44,185,646 clean reads from the PE and RE libraries, respectively. Approximately 99.86% of the total reads were valid for further analysis (Table 1).

#### De novo assembly of sequencing data

**Figure 1:**
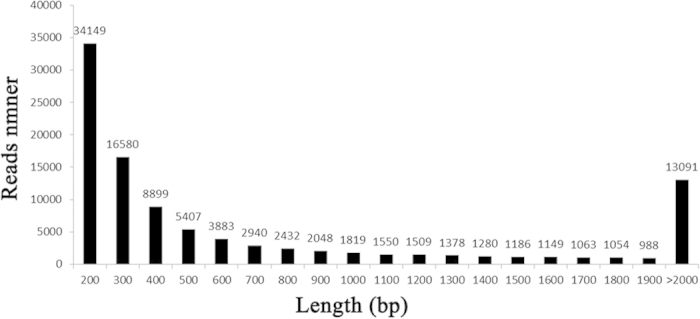
Size distribution of the unigenes.

The Trinity software (http://trinityrnaseq.sourceforge.net/) was used for the *de novo* assembly of our valid reads^26^. The preprocessed sequencing reads were assembled into 102,441 unigenes using the optimized parameters. The assembled unigenes in the present study were evaluated using the following standard metrics: Min length, Median length, Mean length, N50, Max length, and Total length (Table 2). N50 represents a weighted median statistic such that 50% of the entire assembly is contained in unigenes equal to or larger than this value in base pairs. The mean unigene length was no less than 1,874 bp in this study, while the average length of the unigenes was approximately 896 bp. Thus there were 91,787,136 bases were generated and the sequencing depth was about 98× in this study, what fully guaranteed that the low abundance sequence could be detected. The size distribution of the reads is shown in Fig. 1.

#### Unigene Annotation

To exclude interference from alternative splicing of transcripts, first we clustered all of the transcripts that matched the same reference gene; then, we removed redundant transcripts and preserved only the longest transcript from each cluster to represent a unigene^27^. The unigenes were BLASTed to public database banks including SWISS-PROT (a manually annotated and reviewed protein sequence database), nr (NCBI non-redundant protein sequences), KEGG (Kyoto Encyclopedia of Genes and Genomes), KOG (euKaryotic Ortholog Groups), and Pfam (a widely used protein family and structure domain database). The valid reads were assembled into 102,441 unigenes, (Table S1), of which 36,308 (35%) had BLAST hits to known proteins in SWISS-PROT, 15,220 (14.86%) in nr, 29,835 (29.12%) in Pfam, 34,265 (33.45%) in KEGG, and 37,219 (36.33%) in KOG (Table 3). Because one unigene might be annotated to more than one public database, a total of 43,127 coding genes were found after annotation.

**Figure 2:**
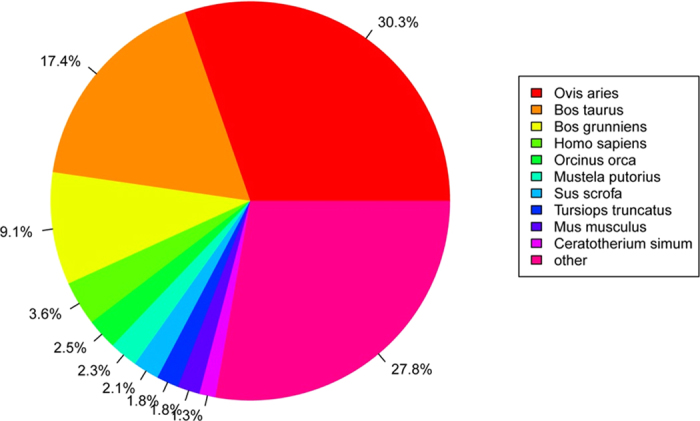
Species distribution of the BLAST hits.

**Figure 3:**
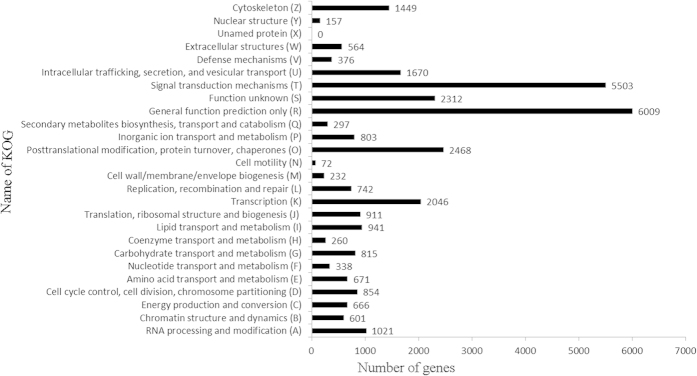
Histogram of unigene KOG classification. The x-axis indicates the number of genes annotation under the group, the y-axis indicates 26 groups of KOG.

To study the sequence conservation of the endometrium in other animal species, we used BLAST^28^ to align the unigenes to the NCBI non-redundant database (nr) using an E-value of e^−10^ as the threshold. A total of 15,220 unique sequences (14.86%) had BLAST hits in the nucleotide sequence database in nr. The majority of the annotated sequences corresponded to known nucleotide sequences of animal species, with 30.3%, 17.4%, 9.1%, 3.6%, and 2.5% matching with *Ovis aries, Bos taurus, Bos grunniens, Homo sapiens*, and *Orcinus* sequences, respectively (Fig. 2). KOG (clusters of orthologous groups for eukaryotic complete genomes) is a classification system based on orthologous genes^29^. In this study, 31,778 unigenes were annotated to 26 groups by the KOG database (Fig. 3). General functional prediction alone (R) annotated 6,009 unigenes at most, and no unigenes were annotated to the Unnamed protein (X). Cell cycle control, cell division, chromosome partitioning (D) was annotated with 854 unigenes, Extracellular structures (W) was annotated with 113 unigenes.

#### Detecting SNP and SSR

Next-generation sequencing provides a range of new potential applications for evolutionary and ecological-genetic studies in non-model species^30^. The discovery of putative SNPs in the RE and PE datasets was summarized. According to the results presented in Table 4, a total of 76,729 putative SNPs were identified for the PE dataset (Table S2), of which 55,044 (71.74%) were transitions and 21,685 (28.26%) were transversions. Similarly, 77,102 putative SNPs were identified from the RE dataset (Table S3), of which 72.13% were transitions and 27.87% were transversions.

**Figure 4:**
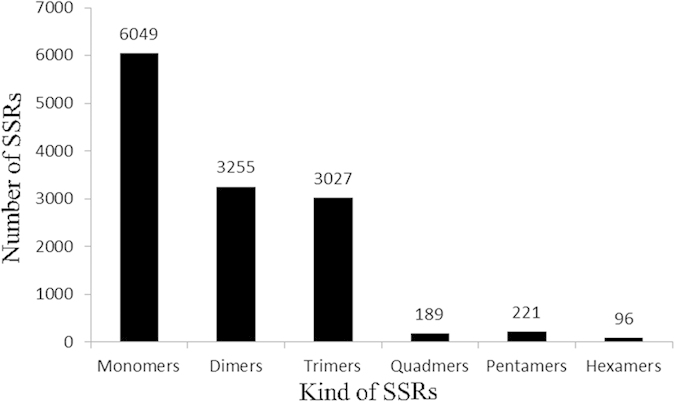
Histogram of the SSRs in pre-receptive and receptive endometrium.

SSRs consist of tandem repeats of short (1–6 bp) nucleotide motifs^31^ that are distributed throughout the genome^32^,^33^. After screening for SSRs in the 102,441 unique sequences using the MISA software, we identified 12,837 SSRs distributed in 10,330 sequences (Table S4). A total of 1,556 sequences contained more than one SSR. Based on the repeat motifs, the SSR loci were divided into monomers (47.12%), dimers (25.36%), trimers (23.58%), quadramers (1.47%), pentamers (1.72%), and hexamers (0.75%) (Fig. 4).

### Differential gene expression and functional characterization

#### Analysis of Unigene Expression

The RNA-Seq technique allowed the analysis of differential expression profiles via transcript abundance with a high sensitivity for transcripts expressed at low levels^34^,^35^. We generated 90 million paired-end reads 100 bp in length, yielding approximately 9 GB of sequence. Thus, the sequencing depth in this study was sufficient to detect transcripts expressed at low levels. To better categorize the unigenes that presented differential expression levels, unigenes expression values RPKM (reads per kilobase of exon model per million mapped reads) were categorized into three groups: high (>500 RPKM), medium (10 to 500 RPKM), and low (<10RPKM) (Table 5). Unigenes that are highly expressed in a specific tissue may be responsible for the basic metabolism and functions of that tissue^12^. A total of 192 and 187 genes were found to be highly expressed in the PE and RE libraries, respectively.

**Figure 5:**
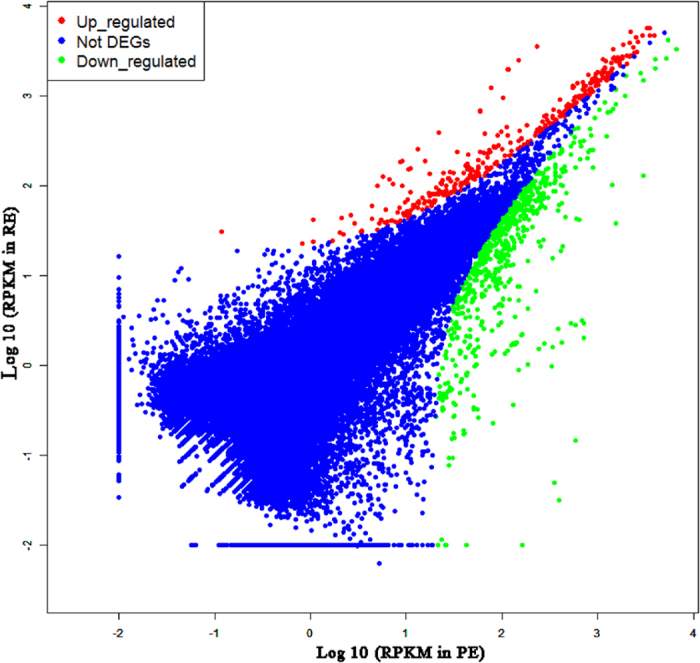
Volcano plots analyses of unigenes in PE and RE. X-axis on both panels shows base 10 logarithm of RPKM of pre-receptive endometrium. Y axis shows receptive endometrium. Red dots indicate up-regulated genes, blue was not DEG, and the green was down-regulated genes in in the receptive endometrium compared to pre-receptive phase.

**Figure 6:**
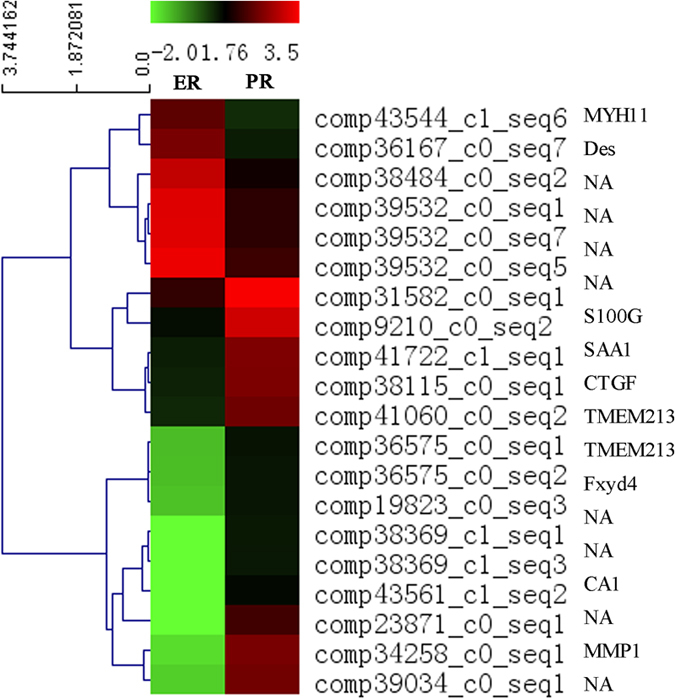
Clustering analysis of differentially expressed unigenes. Heat map of Pearsons correlation across the 20 most differentially expressed transcript tags, and a dendrogram of correlation between transcript tags is shown to the left of the heatmap.

A total of 3,255 unigenes were found to differ significantly in terms of expressional levels (*P* < 0.05) between the PE and RE libraries; the full list of DEGs is provided in Table S5. There were 208 differentially expressed unigenes that were down-regulated in the RE compared to the PE in goats, while 618 unigenes were up-regulated with fold-changes greater than or equal to 2 (Fig. 5). Additionally, there were 5 unigenes specifically expressed in the receptive endometrium with expression values at the medium level. The top 10 unigenes that were up-regulated in RE compared to PE are shown in Table 6. comp34258_c0_seq1 (MMP1) was the most up-regulated DEG (13.61-fold increase in the RE compared to the PE), followed by comp41692_c0_seq2 (MMP12, 10.89-fold) and comp19823_c0_seq3 (Fxyd4, 8.48-fold). The top 10 down-regulated unigenes are shown in Table 7. The most down-regulated DEG was comp24892_c0_seq2 (NNAT, -5.32-fold increase in the RE compared with the PE), followed by comp43544_c1_seq3 (MYH11, -4.41-fold). A heat map of the Pearson’s correlation and a dendrogram of the correlation between transcript tags are provided in Fig. 6. The up-regulated DEG with the highest level of expression (RPKM = 1525.24) was comp9210_c0_seq2 (S100G) with a 5.34-fold increase in the RE, and the down-regulated DEG with the highest level of expression (RPKM = 1985.32) was comp39532_c0_seq7 (Tes) with a -4.09-fold increase in the RE (Table 8).

#### Gene Ontology Analysis of the DEGs

**Figure 7:**
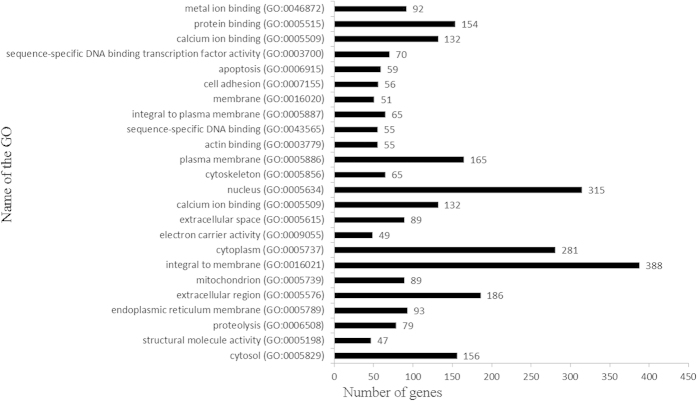
Histogram presentation of the top 20 GO functional annotations for the DEGs. The right x-axis indicates the number of genes in a category. The left y-axis indicates the specific category of GO.

The DEGs were analysed by running queries for each DEG against the GO database, which provides information related to three ontologies: molecular function, cellular component, and biological process. In this study, GO enrichments of the DEGs were categorized into 426 functional groups that met the criteria of *P*-values < 0.001. Out of the 133 terms that were significantly enriched in molecular functions (Table S6), the most significantly enriched GO terms were protein binding (GO: 0005515) with 154 genes annotated, followed by ATP binding (GO: 0005524), calcium ion binding (GO: 0005509), metal ion binding (GO: 0046872), and sequence-specific DNA binding transcription factor activity (GO: 0003700). In the cellular compartment GO category, 88 terms were significantly enriched (Table S7). The most significantly enriched GO terms were integral to membrane (GO: 0016021) with 338 genes annotated, followed by nucleus (GO: 0005634), cytoplasm (GO: 0005737), and extracellular region (GO: 0005576). In the biological processes, 205 GO terms were significantly enriched and were related to various processes (Table S8) such as proteolysis (GO: 0006508), apoptosis (GO: 0006915), cell adhesion (GO: 0007155), protein transport (GO: 0015031), protein folding (GO: 0006457), multicellular organismal development (GO: 0007275), and cell differentiation (GO: 0030154). The top 20 GO functional annotations for the DEGs are shown in Fig. 7. The inclusion of the annotation for insulin-like growth factor binding (GO: 0005520) and clathrin sculpted gamma-aminobutyric acid transport vesicle membrane (GO: 0061202) interested us, because previous studies have reported that IGF-1 (insulin-like growth factor) and GABA (gamma-aminobutyric acid) may play important roles in the development of the receptive endometrium in mice and humans^36^,^37^,^38^,^39^.

#### KEGG Pathway Analysis of the DEGs

Various genes cooperate with each other to exercise their biological functions. Accordingly, KEGG analysis helps us to further understand the biological functions of DEGs^40^. Overall, the DEGs were significantly enriched in 82 KEGG pathways, meeting the criteria of P-values < 0.001 (Table S9), suggesting that these pathways may play important roles in the development of endometrial receptivity.

**Figure 8:**
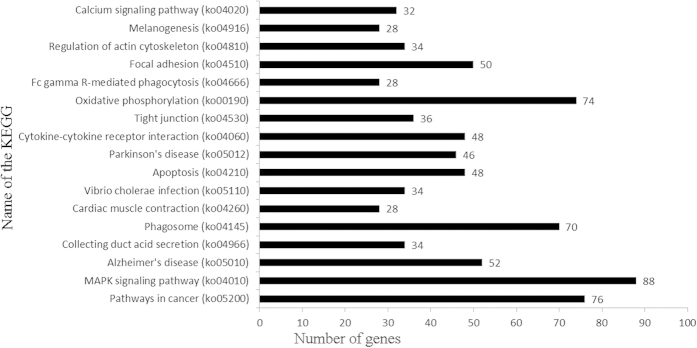
Histogram presentation of the top 17 KEGG functional annotations for the DEGs. The x-axis indicates the number of unique sequences assigned to a specific pathway, the y-axis indicates the KEGG pathway.

The KEGG pathways showing the highest levels of significance were the MAPK signalling pathway (ko04010, 88) with 88 DEGs enriched, followed by Pathways in cancer (ko05200, 76), Oxidative phosphorylation (ko00190, 74), Phagosome (ko04145, 70), Alzheimer’s disease (ko05010, 52), Focal adhesion (ko04510, 50), Cytokine-cytokine receptor interaction (ko04060, 48), and Apoptosis (ko04210, 48). The top 17 KEGG pathways are shown in Fig. 8. These results indicate that diversifying metabolic processes are active in the development of a receptive endometrium from the pre-receptive phase, and a variety of metabolites are synthesized in the receptive endometrium.

#### Genes Possibly Involved in the Development of Receptive Endometria

**Figure 9:**
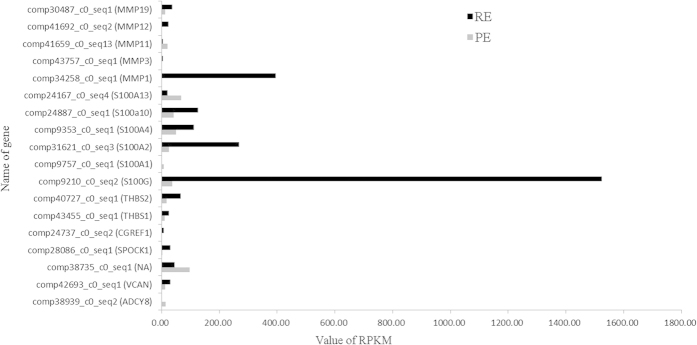
The expression levels of 18 genes exhibited significantly different expression about Calcium and Cell adhesion.

Our analysis identified many DEGs that were enriched in Calcium (GO: 0005509, GO: 0005509, GO: 0008294, and ko04020) and Cell adhesion (GO: 0045785, GO: 0007155, and ko04514) were significant according to the analysis results of both the GO terms and KEGG pathways (*P* < 0.001). Based on these results, we analysed the mRNA expression levels of some coding genes related to calcium and cell adhesion, and found that *ADCY8, VCAN, NA, SPOCK1, CGREF1, THBS1, THBS2, S100G, S100A1, S100A2, S100A4, S100A10, S100A13, MMP1, MMP3, MMP11, MMP12*, and *MMP19* exhibited significantly different expression levels among the two endometrial phases (Fig. 9). Therefore, the results of this study suggested that these genes may play roles in the differential regulation of goat endometrial development during the receptive and pre-receptive phases; however, the validation of this hypothesis needs further study.

## Discussion

Investigating the transcriptome profile of the receptive and pre-receptive endometrium will contribute to our understanding of the biochemical and physiological development of endometrial receptivity during the “window of implantation”. RNA-Seq offers an unprecedented level of sensitivity and high throughput deep sequencing and has been widely used to detect gene expression patterns^10^,^12^. In the present study, large-scale transcriptome data were obtained using Illumina RNA-Seq as the first step of our endeavour to provide clear insight into the molecular mechanism of endometrial receptivity in dairy goats.

### Sequencing Results

#### Summary and validation of RNA-Seq

The RNA-Seq method has previously been widely used in many tissues and at developmental different stages^41^,^42^. Mamo’s research team summarized a large dataset to characterize the transcriptome of the endometrium and thought that most studies had tended to focus on either the conceptus or the maternal endometrium rather than the crosstalk between the two in cattle^43^. Therefore, they examined the global transcriptome profiles of the day 16 bovine conceptus and pregnant endometrial tissues using RNA-Seq, and a total of 133 conceptus ligands that interact with corresponding receptors on the endometrium and 121 endometrium ligands that interact with corresponding receptors on the conceptus were identified^44^. Additionally, Forde and her colleagues determined how low progesterone (P4) affected the endometrial transcriptome in cattle, and identified a panel of genes that were truly regulated in the endometrium by circulating concentrations of P4 *in vivo*^45^.

To characterize the complex transcriptome changes in the endometrium in the course of initial conceptus attachment, the porcine endometrium between Day 14 pregnancy (Attachment Phase) and corresponding cyclic endometrium was performed using Illumina RNA-Seq^46^. Furthermore, the endometrium transcriptome of day 12 pregnant (Preattachment Phase) was also analysed by Samborski^47^. Therefore, an integrated analysis of gene expression changes during these two distinct phases of early pregnancy in the pig was performed and revealed a number of biological processes and pathways that are potentially involved in the establishment of pregnancy in the pig.

Researchers would get some genes that were unidentified previously using RNA-Seq, what provided a useful approach to gain insights in the physiological events. However, current knowledge on the development of the receptive endometrium has been limited. Herein, for the first time we present a complete dataset detailing the transcriptome of the pre-receptive endometrium (PE) and receptive endometrium (RE) in dairy goats using the Illumina RNA-Seq approach to generate a total of 46,514,662 and 44,185,646 clean reads, respectively.

#### De novo assembly of sequencing data

Previous analyses of transcriptomic data always used the Velvet, ABySS, MIRA, Newbler v2.3, Newbler v2.5p, CLC, or TGICL software programmes for assembly^11^,^48^. However, all of these methods rely on aligning reads to a reference genome, and thus are unsuitable for samples with a partial or missing reference genome. Choosing suitable assemblers and parameters is critical to obtain a better assembly performance, and is even more important in transcriptomic studies in non-model organisms^30^. For these reasons, Grabherr presented the Trinity method for the *de novo* assembly of full-length transcripts and evaluated it on samples from fission yeast, mice and the whitefly, whose reference genomes are not yet available^26^. Compared with other transcriptome assemblers, Trinity recovers more full-length transcripts across a broad range of expression levels with a similar sensitivity to methods that rely on genome alignments^26^. Thus, the Trinity approach provides a unified solution for transcriptome reconstruction in any sample, especially in the absence of a reference genome. Thus we choose Trinity (http://trinityrnaseq.sourceforge.net/) to assembly and got 102,441 unigenes.

#### Unigene Annotation

All of the unigenes were BLASTed (Basic Local Alignment Search Tool) to public database banks. A total of 43,127 coding genes were detected after the unigenes were annotated to SWISS-PROT, nr, KEGG, KOG, and Pfam. Additionally, 15,220 genes had BLAST hits in the nucleotide sequence database in nr, of which 4,612 (30.3%) matched with *Ovis aries* and 2,648 (17.4%) with *Bos taurus.* Orthologous genes have the same function and common ancestors^29^, and KOG is a classification system based on orthologous genes. In this study, most of the annotated genes were in the General functional prediction only (R) category, but no genes were annotated to the Unnamed protein (X) category. The dataset we report here is the largest dataset of different genes representing a substantial portion of the endometrial transcriptome, and most likely includes the majority of the genes involved in the sophisticated networks that regulate endometrial receptivity during embryo implantation.

#### Detecting SNP and SSR Variants by RNA-Seq

RNA-seq has been shown to be an efficient method to detect SNP variants on a large scale at the mRNA level^12^. A total of 100,734 SNPs were discovered in the milk from Holstein cattle using RNA-seq and were validated by Sanger sequencing technologies. The results confirmed that RNA-Seq technology is an efficient and cost-effective method to identify SNPs in transcribed regions^49^. In the present manuscript we supplied 76,729 putative SNPs in RE and 77,102 in PE, which will contribute to furthering our understanding of its development and functions.

SSRs have proven to be more reliable than other markers, and the utility of SSRs in genetic studies is well established^30^. The polymorphisms revealed by SSRs result from variations in repeat number, which primarily result from slipped-strand mispairing during DNA replication. Thus, SSRs reveal much higher levels of polymorphism than most other marker systems^50^,^51^. We screened 12,837 SSR loci in the endometrium transcriptome, of which the monomer repeats (47.12%) were the most frequent type.

### Differential gene expression and functional characterization

#### Analysis of Gene Expression

Successful implantation and trophoblast invasion are facilitated by the degradation of the extracellular matrix (ECM) of the endometria/decidua^52^,^53^ by various proteinases, including the matrix metalloproteinases (MMPs) that are capable of degrading the ECM^54^. MMP-2 and MMP-9 represent the best characterized proteases in the context of trophoblast invasion; additionally, MMP-12 was recently described to execute elastolysis during uterine spiral artery remodelling^55^. In our study, 3,255 unigenes meeting the criteria of P < 0.05 were found to differ significantly in expression levels between the RE and PE. The most up-regulated DEG was MMP-1 (comp34258_c0_seq1). MMP-1 is a zinc-containing endopeptidase that plays a key role in physiological and pathological tissue remodelling^56^,^57^,^58^. It was found to be secreted by trophoblast cells *in vitro*^59^, and Berthold found weak MMP-1 immuno-reactivities throughout pregnancy, preferably in the invasive phenotype of the extravillous trophoblast and its ECM^60^.

The up-regulated DEG with the highest expression level was S100G (S100 calcium binding protein G, comp9210_c0_seq2), which has been identified in the uteri of several species^61^,^62^,^63^,^64^. S100G expression was observed in the uterine inter-implantation sites in mice^65^ and endometrial epithelial cells in rats^62^ during early pregnancy. Further study showed that S100G was up-regulated by oestrogen^66^. However, studies have reported that the levels of S100G were higher in the luteal phase than in the follicular phase in the endometrium of pigs^67^. And this study demonstrated higher expression of S100G in the receptive endometrium of dairy goats for the first time. These findings suggested that endometrial S100G expression is regulated in a stage-specific manner during early pregnancy, and that S100G may have a critical role in regulating the development of endometrial receptivity.

In this study, CA1 (Carbonic anhydrase 1, comp43561_c1_seq2) was specifically expressed in the receptive endometrium of dairy goats. It is a member of the CA family^68^ and catalyses the reversible hydration/dehydration of carbon dioxide^69^. CA1 is considered to be a differentiation marker of the colonic mucosa, and the loss of CA1 expression is associated with the disappearance of differentiated epithelial cells^70^. Currently, little is known concerning the relationship between CA1 and endometrial receptivity during the “window of implantation” in dairy goats. Therefore, we propose that CA1 may be negatively involved in the development of the receptive endometrium from the pre-receptive phase in dairy goats; however, this hypothesis needs further exploration.

Additionally, MYH11 (myosin heavy chain 11, comp43544_c1_seq6) was decreased in the RE compared with the PE. MYH11 has been associated with cell migration and adhesion, intracellular transport, and signal transduction^71^. It encodes myosin-11^72^, a major contractile protein that converts chemical energy into mechanical energy through the hydrolysis of ATP^71^. And several studies have shown that myosin plays important roles in cancer^73^,^74^,^75^,^76^ due to its down-regulation^77^,^78^.

Based on these findings, we propose that *S100G*, *CA1*, and *MYH11* may play important role in the development of the receptive endometrium from the pre-receptive phase. However, the molecular mechanism of the above genes in the regulation of endometrial receptivity requires further study.

#### Gene Ontology Analysis of the DEGs

In total, 426 GO terms were assigned to the DEGs meeting the criteria of *P* < 0.001, of which 133 GO terms were categorized into molecular function, 88 GO terms were categorized into cellular component and 205 GO terms were categorized into biological process. Further analyses showed that proteolysis, apoptosis, cell adhesion, protein transport, protein folding, multicellular organismal development, and cell differentiation were enriched significantly in biological processes. Previous studies have reported that increased proteolysis were found in the culture supernatant of endometrial tissues from women with endometriosis^79^. Controlled extracellular proteolysis is a requirement for angiogenesis (new vessel formation) that depended on controlled interactions between cells and the ECM^80^. Apoptosis, referred to as programmed cell death, is a process by which multiple cell types are eliminated during embryogenesis and in fully developed adult multicellular organisms^46^. Apoptosis is an important biological process involved in the cyclic remodelling of the endometrium^27^,^81^. Kokawa’s in situ analysis revealed that cells undergoing apoptosis were scattered in the functional layer of the early proliferative endometrium^82^. Accumulating evidence has suggested that apoptosis helps to maintain cellular homeostasis during the menstrual cycle through the elimination of senescent cells from the functional layer of the uterine endometrium during the late secretory and menstrual phases of the cycle^79^,^82^. Spontaneous apoptosis of eutopic and ectopic endometrial tissues is impaired in women with endometriosis, and this reduced susceptibility to apoptosis might permit the growth, survival and invasion of endometriotic tissue^83^,^84^. The unique cell adhesion of the trophoblast to the endometrial epithelium and the subsequent trophoblastic invasion of the maternal tissue is an essential element of embryo implantation^85^. Genes related to cell adhesion processes are particularly important for implantation and placenta formation, and Samborski reported that related categories were found as overrepresented for genes with higher expression in Day 14 pregnant endometrium as well as for genes with higher expression in Day 14 cyclic endometrium^46^. These represent important and necessary processes for the development of the receptive endometrium from the pre-receptive phase, the folded endometrial epithelial bilayer and the ability of the endometrium to acquire the adhesive properties that allow embryo adhesion and its subsequent invasion.

Additionally, 9 genes were significantly annotated to insulin-like growth factor binding (GO: 0005520). Insulin-like growth factor (IGF-1) mediates the growth-promoting activity of the hormone, induces endothelial cell migration and is involved in the regulation of angiogenesis^86^. Oestrogen has been shown to stimulate IGF-1 gene expression in the endometrium^36^,^87^, and a growing body of evidence supports interactions between oestrogen and the IGF signalling pathways^88^. Giudice *et al.* provided evidence of IGF-I involvement in endometrial growth regulation^89^, thereby promoting endothelial cell proliferation and differentiation^90^.

#### KEGG Pathway Analysis of the DEGs

These KEGG pathways with DEGs enriched provide a valuable resource for investigating specific processes, functions, and pathways, facilitated the identification of novel genes involved in the development of the receptive endometrium in goats during the ‘window of implantation’.

Previous studies have reported that cell cycle pathway can be rapidly elicited by oestradiol (E2) in the human and mouse uterus^91^,^92^,^93^. Progesterone has been found to inhibit endometrial cancer by inhibiting the cell cycle^94^, and Dai found that the number of endometrial cells in G1 is significantly increased following treatment with progesterone^81^. Moreover, a few cell cycle regulatory genes were discovered to be expressed within post-hatching and/or preimplantation blastocysts^27^. In this study, the KEGG pathway of Cell cycle (ko04110) with 22 DEGs enriched meet the criteria of P-values < 0.001. Samborski identified some genes that were more highly expressed at the mRNA level in the endometria of pigs on day 12 of pregnancy, and these genes were related to KEGG functional terms of the cell cycle^47^.

In addition, Mitko reported that a number of mRNAs that encode ECM proteins and components of the cytoskeleton are enriched in the bovine endometrium during the oestrous cycle, indicating a remodelling of the ECM in the endometrium and changes in the cytoskeleton of endometrial cells^84^. The possibility that trophoblasts are scavengers of endometrial ECM breakdown products is compelling, and the analyses of ECM receptors and associated molecules that may be present on the trophoblast cell membrane adjacent to these structures will shed light on their precise nature and function^82^. The levels of a number of molecules have been shown to peak during the “window of implantation”, particularly the ECM receptor integrin αvβ3^95^. We found 24 DEGs enriched into the pathway of ECM-receptor interaction (ko04512), met the criteria of P-values < 0.001. Furthermore, temporally and spatially regulated changes in the expression of ECM molecules at the maternal-foetal interface during embryo implantation are crucial for blastocyst attachment, migration and invasion^96^.

#### Potential genes involved in the development of the receptive endometrium

In this study, we analyzed the enriched GO terms and KEGG pathways and found Calcium and Cell adhesion what aroused our interest based on the researches of predecessors. So we filtered the GO-terms and KEGG pathways related to Calcium (GO: 0005509, GO: 0005509, GO: 0008294, and ko04020) and Cell adhesion (GO: 0045785, GO: 0007155, and ko04514), what were significantly enriched for the DEGs from the receptive and pre-receptive endometria.

Large-conductance calcium-activated potassium channels (BκCa channels) were expressed in endometrial cells, affected embryo implantation by mediating endometrial receptive factors, and altered the activity of NF-κB and homeostasis of Ca^2+^ in the human endometrial cells^97^. S100A11 was a Ca^2+^-binding protein that interpreted the calcium fluctuations and elicited various cellular responses. Endometrial S100A11 was a crucial intermediator in EGF-stimulated embryo adhesion, endometrium receptivity, and immunotolerance via affecting Ca^2+^ uptake and released from intracellular Ca^2+^ stores, and down-regulation of S100A11 might cause reproductive failure^98^. In addition, Membrive reported that Calcium could potentiate the effect of estradiol on PGF2α production in the bovine endometrium^99^. Cell-cell adhesion between the conceptus trophectoderm and endometrial luminal epithelial cells is the final step for placentation, and various cell adhesion molecules are involved in this process^100^. The expression and function of cell adhesion molecules are very important for embryo implantation and the establishment of pregnancy^101^. What’s more, LAY reported that interleukin 11 regulated endometrial cancer cell adhesion and migration via STAT3^102^, and L1 cell adhesion molecule is a strong predictor for distant recurrence and overall survival in early stage endometrial cancer^103^.

Therefore, GO terms and/or KEGG pathways that were closely related to calcium and cell adhesion in our study provided much valuable information for future study of the molecular mechanism underlying the development of the receptive endometrium in goats. And then we analysed the mRNA expression levels of some putative coding genes and found that *ADCY8, VCAN, SPOCK1, THBS1* and *THBS2* were involved in the GO terms and KEGG pathways related to Calcium and Cell adhesion.

ADCY8 (adenylate cyclase 8) is a membrane-bound enzyme that catalyses the formation of cAMP from ATP in higher vertebrates^104^ after it is activated by calmodulin^105^,^106^. Previous findings showed that ADCY8 is required for axonal pathfinding before axons reach their targets, connecting mouse avoidance behaviour obtained in automated home cage environments to human bipolar affective disorder^107^. The findings in the present study point to novel mechanisms underlying the role of the regulatory factor in the formation of the receptive endometrium. VCAN is a chondroitin sulphate proteoglycan that was first identified in fibroblastic extracts^108^ and is abundantly expressed within the ECM of the developing and mature cardiovascular system^109^. Since the initial identification and characterization of VCAN, the functional importance of this chondroitin sulphate proteoglycan in multiple adult biological systems has been increasingly demonstrated^110^,^111^. Additionally, because it interacts directly with integrin, attachment-dependent signalling may be altered and affect cell migration and the epithelial mesenchymal transition in the cushion primordia of the septa and valves^112^,^113^. SPOCK1 (SPARC/osteonectin, cwcv and kazal-like domains proteoglycan 1) encodes testican-1, which is a proteoglycan belonging to the BM-40/SPARC/osteonectin family of extracellular calcium-binding proteins; SPOCK1 is widely expressed in the heart, blood and cartilage^114^,^115^,^116^,^117^. Miao identified SPOCK1 as a novel transforming growth factor-b1 (TGF-b) target gene that regulates the lung cancer cell epithelial-to-mesenchymal transition (EMT), which plays a key role in the early process of metastasis of cancer cells^118^. Moreover, a number of studies have demonstrated that SPOCK1 plays a critical role in prostate cancer recurrence and glioblastoma invasion^119^,^120^. Thus, we hypothesize that SPOCK1 plays an important role in the development of the receptive endometrium, although this hypothesis needs further study. THBS1 (thrombospondin 1) is a multifunctional glycoprotein that is involved in numerous biological processes, such as cellular adhesion, angiogenesis, metastasis, inflammation, atherosclerosis, homeostasis^121^,^122^. THBS1 is generally considered to be a tumour suppressor and mediates cell-to-cell and cell-to-matrix interactions that are important for platelet aggregation and angiogenesis^123^,^124^. THBS2 (Thrombospondin 2) is an ECM disulphide-linked homotrimeric glycoprotein^95^ that plays an important role in cell adhesion, migration and proliferation, cell-to-cell and cell-to-matrix interactions, and angiogenesis^125^. It has also been implicated in atherosclerosis and thrombosis due to its function in the regulation of matrix metallopeptidase 2 (MMP2)^126^,^127^. Thus, THBS1 and THBS2 may play a major role in ECM homeostasis. However, only very limited data is available concerning the status of THBS1 and THBS2 in the development of the receptive endometrium, what needs further study.

In conclusion, we sequenced the transcriptome of dairy goats with the Illumina 2500 sequencing platform. Trinity software was used for *de novo* assembly of our valid reads. A total of 3,255 DEGs were discovered between the pre-receptive endometrium (PE) and the receptive endometrium (RE), with a threshold of P < 0.05. Based on the results of GO and KEGG enrichment, some genes potentially play an important role in the development of endometrial receptivity. Additionally, 76,729–77,102 putative SNPs and 12,837 SSRs were discovered in this study. Our results not only reveal new information regarding the development of dairy goat endometrial receptivity but also provide a broad and novel vision for future research at the molecular level in dairy goats.

## Methods

### Ethics statement

All animals in this study were maintained according to the No. 5 proclamation of the Ministry of Agriculture, P. R. China. And animal protocols were approved by the Review Committee for the Use of Animal Subjects of Northwest A&F University.

### Study Design and Sample Collection

A total of 20 healthy, 24-month-old multiparous dairy goats (Xinong Saanen) were induced to oestrous synchronization for this study. The first day of mating was considered to be day 0 of pregnancy. Gestational days 5 and 15 are important time points for embryo implantation in goats^128^. The experimental goats were observed 3 times daily to ascertain oestrous signs and mated naturally twice during oestrus. Then, the goats were euthanized following intravenous injection of a barbiturate (30 mg/kg) at gestational day 5 (pre-receptive endometrium) and gestational day 15 (receptive endometrium). Endometrium samples from 10 goats at gestational day 5 and 10 goats at gestational day 15 were obtained from the anterior wall of the uterine cavity. All tissue samples were washed briefly with PBS (Phosphate Buffered Saline) and then immediately frozen in liquid nitrogen.

### Library Construction and Sequencing

Total RNA was extracted from every sample using the TRIzol reagent (Invitrogen, CA, USA) and DNA contamination was evaluated using DNase (TaKaRa, Dalian, China). The total RNA quantity and purity were analysis of Bioanalyzer 2100 (Agilent, CA, USA) and RNA 6000 Nano LabChip Kit (Agilent, CA, USA) with RIN number > 7.0. The total RNA with lowest quality was not used for further study from the pre-receptive endometrium samples and receptive endometrium samples, respectively. Approximately 5 ug of total RNA was subjected to isolate Poly (A) mRNA with poly-T oligo attached magnetic beads (Invitrogen). Following purification, the mRNA was fragmented into small pieces using divalent cations under elevated temperature. Then the cleaved RNA fragments were reverse-transcribed to create the final cDNA library in accordance with the protocol for the mRNA-Seq sample preparation kit (Illumina, San Diego, USA). Each PE and RE library was constructed by pooling 9 homogenized total RNAs from the endometrium samples. Then, paired-end sequencing of the PE and RE libraries was performed on the Illumina Hiseq2500 (LC Sciences, USA) following the vendor’s recommended protocol. The length of the reads was 100 bp, and the average insert size for the paired-end libraries was 179 bp (the length of the adapter was 121 bp).

### Primary Analysis and Sequence Assembly

Prior to assembly, raw reads (raw data) in the fastq format were processed using previously described scripts^29^. In this step, clean reads (valid data) were obtained by removing reads containing adapter sequences, ploy-N and the sequencing primer as well as low quality reads (1, reads containing sequencing adaptors; 2, reads containing sequencing primer; 3, nucleotide with q quality score lower than 20) from the raw data. At the same time, Q20, Q30, GC-content and sequence duplication level of the clean data were calculated. All of the downstream analyses were based on high quality clean data. On the basis of previousresearch work^26^,^36^, the publicly available program Trinity (http://trinityrnaseq.source forge.net/) was selected for assembly in this study and used to calculate the N50 number, average length, max length and contig number during different length intervals.

### Sequence Functional Annotation

Unigene annotations provide functional annotations for all unigenes in addition to their expression levels. Functional annotations of unigenes were analysed using protein sequence similarity by LC-Bio (Hangzhou, China). Protein function information could be predicted from annotations of the most similar proteins in the following published databases: SWISS-PROT (A manually annotated and reviewed protein sequence database, ftp://ftp.uniprot.org/pub/databases/uniprot/currentrelease/knowledgebase/complete/uniprot sprot.fasta.gz), NR (NCBI non-redundant protein sequences, ftp://ftp.ncbi.nlm.nih.gov/ blast/db/FASTA/nr.gz), KEGG (Kyoto Encyclopedia of Genes and Genomes, http://www. kegg.jp/kegg/download/), KOG (Karyotic Ortholog Groups, http://www.ncbi.nlm.nih.gov/ COG/grace/shokog.cgi), and Pfam (A widely used protein family and structure domain database, ftp://ftp.sanger.ac.uk/pub/databases/Pfam/releases/Pfam27.0/Pfam-A.fasta.gz). All searches were conducted with BLASTx (http://blast.ncbi.nlm. nih.gov/) using a minimum E-value of < 1e-10 as the threshold^29^,^129^.

### Analysis of Gene Expression

To investigate the expression level of each unigene in different samples, all unigenes for each sample were aligned back to the final assembly using Perl scripts in Trinity under the default parameters option. The alignment produced digital expression levels for each contig that were normalized with a RESM-based algorithm using Perl scripts in the Trinity package to obtain RPKM values. Gene expression levels were normalized by considering the RPKM value (reads per kilobase of the exon model per million mapped reads) and the effect of the sequencing depth and gene length on the read counts; this approach represents the most commonly used method for estimating gene expression levels^25^. The fold changes (log2 (RE_RPKM_/PE_RPKM_)) and the corresponding significance threshold of the P-value were estimated according to the normalized gene expression level. Based on the expression levels, the significant DEGs between PE and RE were identified with “P < 0.05 and |log_2_ fold change|>1” used as the threshold to judge the DEGs in this study.

### Gene ontology and pathway enrichment analysis of DEGs

Gene ontology (GO) is an international standard gene functional classification system^130^. The hypergeometric test was applied to map all differentially expressed genes to terms in the GO database^131^. The corrected P-value < 0.001 was used as the threshold to find significantly enriched GO terms in the input list of DEGs compared to their genomic background. The formula of P-value was as follow (numbered 1):


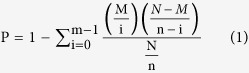


The N represented the number of GO annotated genes in genome, n represented the number of differentially expressed genes in N. M represented the number of particular GO annotated genes in genome, m represented the number of particular GO annotated genes expressed differentially in M^132^. And then P was less than 0.001 was used as the threshold to judge significant enrichment GO term in this study^10^,^12^.

KEGG is the major public pathway-related database that helps to further understanding the biological functions of genes with high level functions and the utilities of the biological system from large-scale molecular datasets (http://www.genome.jp/kegg/)^29^. Pathway enrichment analysis identifies significantly enriched metabolic pathways or signal transduction pathways^10^ using the corrected P-value < 0.001 as a threshold to find significantly enriched KEGG terms in the input list of DEGs compared to their genomic background^133^. The calculation formula is the same as that used for the GO analysis.

### Detection of Putative SNP and SSR

High-throughput SNP data has demonstrated its potential for making inferences about demographic and adaptive processes in natural populations^134^,^135^,^136^,^137^. The SNPs of PE and RE at the transcriptome level were analysed based on the massively parallel Illumina technology. The Bowtie (http://bowtie-bio.sourceforge.net/) and Samtools (http://samtools.sourceforge. net/) software programmes were used to identify SNPs; all reads were included in this process^129^. The sample data were mapped to the transcript with the Bowtie software after pretreatment based on the transcription library. Further SNP analysis was performed based on the results of mapping, and variable sites with higher possibilities were further filtered using the software Samtools.

The MISA (Microsatellite) (http://pgrc.ipkgatersleben.de/misa) was used to identify SSRs. The Batch Primer3 program was used to design primer pairs for amplification of the SSR motifs^138^. The default settings were used with the exception of the annealing temperature, which was set for an optimum of 60 °C^129^. Monomers, dimers, trimers, quadramers, pentamers and hexamers were all considered as the search criteria for SSRs in the MISA script.

## Additional Information

**Accession codes**: All the basic data series were submitted to NCBI's Sequence Read Archive with accession number SRP056134

**How to cite this article**: Zhang, L. *et al.* Characterization of the Transcriptional Complexity of the Receptive and Pre-receptive Endometria of Dairy Goats. *Sci. Rep.* **5**, 14244; doi: 10.1038/srep14244 (2015).

## Supplementary Material

Table S1

Table S2

Table S3

Table S4

Table S5

Table S6

Table S7

Table S8

Table S9

Supporting Information

## Figures and Tables

**Table 1:** Overview of the sequencing reads and reads after preprocessing.

Sample	Raw Reads	Raw Bases	Clean Reads	Clean Bases	Valid Ratio(Reads)%
Pre-receptive endometrium (PE)	46,582,140	465,8214,000	46,514,662	4,651,466,200	99.86%
Receptive endometrium (RE)	44,249,664	442,4966,400	44,185,646	4,418,564,600	99.86%

**Table 2:** Assembly results of unigenes.

Item	Unigene	Min length	Median length	Mean length	N50	Max length	Total length
Number	102,441	201	404	896	1,874	13,764	91,874,680

**Table 3:** Annotation result statistics of unigenes in different databases.

Databases	SWISS-PROT	NR	Pfam	KEGG	KOG
Number of Unigenes	36308	15220	29835	34265	37219
Percent (%)	35.44%	14.86%	29.12%	33.45%	36.33%

Note: SWISS-PROT, a manually annotated and reviewed protein sequence database.

NR, NCBI non-redundant protein sequences.

Pfam, a widely used protein family and structure domain database.

KEGG, Kyoto Encyclopedia of Genes and Genomes.

KOG, Karyotic Ortholog Groups.

**Table 4:** Result statistics of putative SNPs in pre-receptive and receptive endometrium.

SNP type	RE library/n	PE library/n
Transition	55,044	55,611
A-G	27,339	27,783
C-T	27,705	27,828
Transversion	21,685	21,491
A-C	5,489	5,453
A-T	3,695	3,581
C-G	6,796	6,813
G-T	57,055	5,644
Total	76,729	77,102

**Table 5:** RNA-Seq gene expression results for the RE and PE libraries (RPKM).

Category	PE	RE
AHighly expressed genes	192	187
BMedium expressed genes	7,855	8,253
CLowly expressed genes	89,507	87,677
Total expressed genes	97,544	96,177
Unexpressed genes	4,885	6,322

Note: RPKM, Reads Per Kilobase of exon model per Million mapped reads.

^A^Gene expressional level > 500 RPKM.

^B^Gene expressional level ranging from 10 to 500 RPKM.

^C^Gene expressional level < 10 RPKM.

**Table 6:** The top 10 unigenes that up-regulated in RE compared with PE.

ID	Annotated gene	Length	PE (RPKM)	RE (RPKM)	log2 (RE/PE)	P-value
comp24368_c0_seq2	NTS	1366	2.04	718.84	8.46	0
comp39034_c0_seq2	/	604	0.15	582.48	11.97	0
comp34258_c0_seq1	MMP1	1581	0.03	396.20	13.61	1.9126E-88
comp39034_c0_seq1	/	505	0.05	348.97	12.78	4.14E-78
comp28945_c0_seq2	/	331	0.98	326.77	8.38	1.1979E-72
comp37557_c0_seq2	/	1591	0.36	129.13	8.48	8.7967E-30
comp43084_c0_seq85	ATP6V1C2	993	0.23	79.62	8.45	5.5951E-19
comp36575_c0_seq1	TMEM213	1063	0.09	30.22	8.33	4.2434E-08
comp19823_c0_seq3	Fxyd4	485	0.08	27.68	8.48	1.5424E-07
comp41692_c0_seq2	MMP12	2147	0.01	23.58	10.98	1.1715E-06

Note: Annotated gene displayed that the unigenes had the BLAST hits to known proteins in SWISS-PROT, and “/” meant the unigenes were not blasted to SWISS-PROT. Length represented the bases number of sequence. PE (RPKM) represented the RPKM value (Reads Per Kilobase of exon model per Million mapped reads) in the PE library generated from pre-receptive endometrium of Xinong Saanen dairy goats. RE (RPKM) represented the RPKM value in the RE library generated from receptive endometrium of Xinong Saanen dairy goats. log2 (R/P) indicated the fold change between libraries. *P*-Value manifested the significance of DEG between two samples.

**Table 7:** The top 10 unigenes that down-regulated in RE compared with PE.

ID	Annotated gene	Length	PE (RPKM)	RE (RPKM)	log2 (RE/PE)	*P*-value
comp43544_c1_seq6	MYH11	1982	255.96	13.18	−4.28	2.17E-49
comp43544_c1_seq15	MYH11	6602	187.12	9.03	−4.37	6.39E-37
comp43544_c1_seq3	MYH11	6620	183.74	8.62	−4.41	1.99E-36
comp36167_c0_seq5	/	1147	124.63	5.74	−4.44	2.63E-25
comp43563_c1_seq13	MYH11	2176	98.86	5.05	−4.29	4.04E-20
comp24892_c0_seq2	Neuronatin	1298	42.31	1.06	−5.32	4.01E-10
comp37477_c0_seq1	PTN	1710	44.25	2.10	−4.39	6.42E-10
comp43151_c1_seq1	/	859	31.17	0.12	−8.04	2.98E-08
comp16941_c0_seq1	/	201	23.79	1.08	−4.47	5.43E-06
comp34646_c0_seq1	/	1933	22.85	0.82	−4.80	6.20E-06

Note: Annotated gene displayed that the unigenes had the BLAST hits to known proteins in SWISS-PROT, and “/” meant the unigenes were not blasted to SWISS-PROT. Length represented the bases number of sequence. PE (RPKM) represented the RPKM value (Reads Per Kilobase of exon model per Million mapped reads) in the PE library generated from pre-receptive endometrium of Xinong Saanen dairy goats. RE (RPKM) represented the RPKM value in the RE library generated from receptive endometrium of Xinong Saanen dairy goats. log2 (R/P) indicated the fold change between libraries. *P*-Value manifested the significance of DEG between two samples.

**Table 8:** DEG with highly expressed level in R or PE libraries (|log2 (RE/PE)|>2).

ID	Annotated gene	Length	PE (RPKM)	RE (RPKM)	log2 (RE/PE)	P-value
comp25223_c0_seq1	/	841	3492.60	230.13	−3.92	0.00
comp42940_c0_seq4	/	3052	671.00	59.12	−3.50	0.00
comp39532_c0_seq1	Tes	1456	1958.36	115.02	−4.09	0.00
comp39532_c0_seq7	Tes	1701	1985.32	116.82	−4.09	0.00
comp42901_c0_seq4	IGFBP3	4158	951.25	102.93	−3.21	0.00
comp38484_c0_seq2	/	1744	1241.57	76.95	−4.01	0.00
comp39532_c0_seq5	/	1014	2487.73	149.24	−4.06	0.00
comp42940_c0_seq3	/	3023	676.00	59.41	−3.51	0.00
comp24368_c0_seq2	NTS	1366	2.04	718.84	8.46	0.00
comp29997_c0_seq1	COL1A2	5188	42.26	634.41	3.91	0.00
comp42978_c0_seq2	COL3A1	5664	159.37	884.09	2.47	0.00
comp25409_c0_seq1	PLA2G10	812	3.13	687.16	7.78	0.00
comp30511_c0_seq1	/	1189	42.98	594.48	3.79	0.00
comp9210_c0_seq2	S100G	495	37.68	1525.24	5.34	0.00
comp36285_c0_seq1	/	1302	101.33	1400.23	3.79	0.00
comp39911_c0_seq1	S100G	1060	2.98	709.07	7.90	0.00
comp39034_c0_seq2	/	604	0.15	582.48	11.97	0.00
comp39911_c0_seq8	S100G	1150	2.84	579.32	7.67	0.00
comp25409_c0_seq5	PLA2G10	902	2.94	523.92	7.48	0.00
comp31582_c0_seq1	/	2192	128.19	2994.99	4.55	0.00
comp23871_c0_seq1	/	1179	0	163.62	Inf	1.39E-37
comp43561_c1_seq2	CA1	2100	0	42.20	Inf	7.67E-11
comp38369_c1_seq3	/	563	0	25.83	Inf	3.57E-07
comp38369_c1_seq1	/	457	0	25.28	Inf	4.74E-07
comp38369_c1_seq5	/	680	0	21.24	Inf	3.92E-06

Note: Annotated gene displayed that the unigenes had the BLAST hits to known proteins in SWISS-PROT, and “/” meant the unigenes were not blasted to SWISS-PROT. Length represented the bases number of sequence. PE (RPKM) represented the RPKM value (Reads Per Kilobase of exon model per Million mapped reads) in the PE library generated from pre-receptive endometrium of Xinong Saanen dairy goats. RE (RPKM) represented the RPKM value in the RE library generated from receptive endometrium of Xinong Saanen dairy goats. log2 (R/P) indicated the fold change between libraries. *P*-Value manifested the significance of DEG between two samples.

## References

[b1] Xia H. F., Jin X. H., Cao Z. F., Hu Y. & Ma X. MicroRNA expression and regulation in the uterus during embryo implantation in rat. FEBS J. 281, 1872–1891, 10.1111/febs.12751 (2014). 24528955

[b2] Egashira M. & Hirota Y. Uterine receptivity and embryo–uterine interactions in embryo implantation: lessons from mice. Reproductive Medicine and Biology 12, 127–132 (2013). 10.1007/s12522-013-0153-1 PMC5906852 29699140

[b3] Simmons D. G. & Kennedy T. G. Uterine sensitization-associated gene-1: A novel gene induced within the rat endometrium at the time of uterine receptivity/sensitization for the decidual cell reaction. Biology of Reproduction 67, 1638–1645, 10.1095/bioreprod.102.006858 (2002). 12390898

[b4] Revel A., Achache H., Stevens J., Smith Y. & Reich R. MicroRNAs are associated with human embryo implantation defects. Human reproduction, 26, 2830–2840, 10.1093/humrep/der255 (2011). 21849299

[b5] Pan Q., Luo X., Toloubeydokhti T. & Chegini N. The expression profile of micro-RNA in endometrium and endometriosis and the influence of ovarian steroids on their expression. Mol. Human Reprod. 13, 797–806, 10.1093/molehr/gam063 (2007). 17766684

[b6] Altmäe S. *et al.* MicroRNAs miR-30b, miR-30d, and miR-494 regulate human endometrial receptivity. Reproductive Sciences 20, 308–317 (2013). 22902743 10.1177/1933719112453507 PMC4077381

[b7] Boivin J., Bunting L., Collins J. A. & Nygren K. G. International estimates of infertility prevalence and treatment-seeking: potential need and demand for infertility medical care. Human reproduction 22, 1506–1512 (2007). 17376819 10.1093/humrep/dem046

[b8] Macklon N. S., Stouffer R. L., Giudice L. C. & Fauser B. C. The science behind 25 years of ovarian stimulation for *in vitro* fertilization. Endocr. Rev. 27, 170–207 (2006). 16434510 10.1210/er.2005-0015

[b9] Sha A.-G. *et al.* Genome-wide identification of micro-ribonucleic acids associated with human endometrial receptivity in natural and stimulated cycles by deep sequencing. Fertility and sterility 96, 150–155. e155 (2011). 21601191 10.1016/j.fertnstert.2011.04.072

[b10] He H. & Liu X. Characterization of Transcriptional Complexity during Longissimus Muscle Development in Bovines Using High-Throughput Sequencing. PloS one 8, e64356 (2013). 23762238 10.1371/journal.pone.0064356 PMC3676445

[b11] Kumar S. & Blaxter M. L. Comparing de novo assemblers for 454 transcriptome data. BMC Genomics 11, 571 (2010). 20950480 10.1186/1471-2164-11-571 PMC3091720

[b12] Zhou Y. *et al.* Characterization of Transcriptional Complexity during Adipose Tissue Development in Bovines of Different Ages and Sexes. PloS one 9, e101261 (2014). 24983926 10.1371/journal.pone.0101261 PMC4077742

[b13] Cloonan N. & Grimmond S. M. Transcriptome content and dynamics at single-nucleotide resolution. Genome Biol 9, 234 (2008). 18828881 10.1186/gb-2008-9-9-234 PMC2592708

[b14] Li H., Ruan J. & Durbin R. Mapping short DNA sequencing reads and calling variants using mapping quality scores. Genome Res. 18, 1851–1858 (2008). 18714091 10.1101/gr.078212.108 PMC2577856

[b15] Nagalakshmi U. *et al.* The transcriptional landscape of the yeast genome defined by RNA sequencing. Science 320, 1344–1349 (2008). 18451266 10.1126/science.1158441 PMC2951732

[b16] Sultan M. *et al.* A global view of gene activity and alternative splicing by deep sequencing of the human transcriptome. Science 321, 956–960 (2008). 18599741 10.1126/science.1160342

[b17] Wilhelm B. T. *et al.* Dynamic repertoire of a eukaryotic transcriptome surveyed at single-nucleotide resolution. Nature 453, 1239–1243 (2008). 18488015 10.1038/nature07002

[b18] AC’t Hoen P. *et al.* Deep sequencing-based expression analysis shows major advances in robustness, resolution and inter-lab portability over five microarray platforms. Nucleic Acids Res. 36, e141–e141 (2008). 18927111 10.1093/nar/gkn705 PMC2588528

[b19] Marioni J. C., Mason C. E., Mane S. M., Stephens M. & Gilad Y. RNA-seq: an assessment of technical reproducibility and comparison with gene expression arrays. Genome Res. 18, 1509–1517 (2008). 18550803 10.1101/gr.079558.108 PMC2527709

[b20] Xia H.-F. *et al.* Temporal and spatial regulation of miR-320 in the uterus during embryo implantation in the rat. International journal of molecular sciences 11, 719–730 (2010). 20386663 10.3390/ijms11020719 PMC2852863

[b21] Venners S. A. *et al.* Urinary estrogen and progesterone metabolite concentrations in menstrual cycles of fertile women with non-conception, early pregnancy loss or clinical pregnancy. Human Reproduction 21, 2272–2280 (2006). 16798842 10.1093/humrep/del187

[b22] Yoshinaga K. Review of factors essential for blastocyst implantation for their modulating effects on the maternal immune system in Seminars. Semin. Cell Dev. Biol. 19, 161–169 (2008). 18054836 10.1016/j.semcdb.2007.10.006

[b23] Sun Q.-H., Peng J.-P., Xia H.-F., Yang Y. & Liu M.-L. Effect on expression of RT1-A and RT1-DM molecules of treatment with interferon-γ at the maternal—fetal interface of pregnant rats. Human Reproduction 20, 2639–2647 (2005). 15946996 10.1093/humrep/dei105

[b24] Roberts C. T. *et al.* Circulating insulin-like growth factor (IGF)-I and IGF binding proteins-1 and-3 and placental development in the guinea-pig. Placenta 23, 763–770, 10.1053/plac.2002.0849 (2002). 12398816

[b25] Mortazavi A., Williams B. A., McCue K., Schaeffer L. & Wold B. Mapping and quantifying mammalian transcriptomes by RNA-Seq. Nat. Methods 5, 621–628 (2008). 18516045 10.1038/nmeth.1226 PMC13303166

[b26] Grabherr M. G. *et al.* Full-length transcriptome assembly from RNA-Seq data without a reference genome. Nat. Biotechnol. 29, 644–652 (2011). 21572440 10.1038/nbt.1883 PMC3571712

[b27] Wood D. & Levison D. Atrophy and apoptosis in the cyclical human endometrium. The Journal of pathology 119, 159–166 (1976). 956955 10.1002/path.1711190305

[b28] Altschul S. F. *et al.* Gapped BLAST and PSI-BLAST: a new generation of protein database search programs. Nucleic Acids Res. 25, 3389–3402 (1997). 9254694 10.1093/nar/25.17.3389 PMC146917

[b29] Guo Q. *et al.* De novo transcriptome sequencing and digital gene expression analysis predict biosynthetic pathway of rhynchophylline and isorhynchophylline from Uncaria rhynchophylla, a non-model plant with potent anti-alzheimer’s properties. BMC Genomics 15, 676 (2014). 25112168 10.1186/1471-2164-15-676 PMC4143583

[b30] Zhou Y., Gao F., Liu R., Feng J. & Li H. De novo sequencing and analysis of root transcriptome using 454 pyrosequencing to discover putative genes associated with drought tolerance in Ammopiptanthus mongolicus. BMC Genomics 13, 266 (2012). 22721448 10.1186/1471-2164-13-266 PMC3407029

[b31] Gupta P., Balyan H., Sharma P. & Ramesh B. Microsatellites in plants: a new class of molecular markers. Curr. Sci. 45, 45–54 (1996).

[b32] Noormohammadi Z., Trujillo I., Belaj A., Ataei S. & Hosseini-Mazinan M. Genetic structure of Iranian olive cultivars and their relationship with Mediterranean’s cultivars revealed by SSR markers. Scientia Horticulturae 178, 175–183, 10.1016/j.scienta.2014.08.002 (2014).

[b33] Xing R. *et al.* Genetic diversity and population structure of Armillaria luteovirens (Physalacriaceae) in Qinghai-Tibet Plateau revealed by SSR markers. Biochem. Syst. Ecol. 56, 1–7, 10.1016/j.bse.2014.04.006 (2014).

[b34] Robinson M. D. & Oshlack A. A scaling normalization method for differential expression analysis of RNA-seq data. Genome Biol 11, R25 (2010). 20196867 10.1186/gb-2010-11-3-r25 PMC2864565

[b35] Werner T. Next generation sequencing in functional genomics. Briefings in bioinformatics 11, 499–511 (2010). 20501549 10.1093/bib/bbq018

[b36] Rutanen E.-M. Insulin-like growth factors and insulin-like growth factor binding proteins in the endometrium. Effect of intrauterine levonorgestrel delivery. Human Reproduction 15, 173–181 (2000). 11041233 10.1093/humrep/15.suppl_3.173

[b37] Quezada M. *et al.* Proenkephalin A and the γ-aminobutyric acid A receptor π subunit: expression, localization, and dynamic changes in human secretory endometrium. Fertility and sterility 86, 1750–1757 (2006). 17074347 10.1016/j.fertnstert.2006.05.033

[b38] Eyster K. M., Klinkova O., Kennedy V. & Hansen K. A. Whole genome deoxyribonucleic acid microarray analysis of gene expression in ectopic versus eutopic endometrium. Fertility and sterility 88, 1505–1533 (2007). 17462640 10.1016/j.fertnstert.2007.01.056

[b39] Sadeghi H. & Taylor H. S. HOXA10 regulates endometrial GABAA π receptor expression and membrane translocation. American Journal of Physiology-Endocrinology and Metabolism 298, E889–E893 (2010). 20103740 10.1152/ajpendo.00577.2009 PMC3774337

[b40] Kanehisa M. *et al.* Data, information, knowledge and principle: back to metabolism in KEGG. Nucleic Acids Res. 42, D199–D205 (2014). 24214961 10.1093/nar/gkt1076 PMC3965122

[b41] Wickramasinghe S., Rincon G., Islas-Trejo A. & Medrano J. F. Transcriptional profiling of bovine milk using RNA sequencing. BMC Genomics 13, 45 (2012). 22276848 10.1186/1471-2164-13-45 PMC3285075

[b42] Medrano J. *et al.* Comparative analysis of bovine milk and mammary gland transcriptome using RNA-Seq*. 9th World Congress of Genetics Applied to Livestock Production (WCGALP),* Leipzig-Germany, No. 0852 (2010).

[b43] Mamo S., Rizos D. & Lonergan P. Transcriptomic changes in the bovine conceptus between the blastocyst stage and initiation of implantation. Animal Reproduction Science 134, 56–63, 10.1016/j.anireprosci.2012.08.011 (2012). 22944169

[b44] Mamo S., Mehta J. P., Forde N., McGettigan P. & Lonergan P. Conceptus-Endometrium Crosstalk During Maternal Recognition of Pregnancy in Cattle. Biology of Reproduction 87, 10.1095/biolreprod.112.099945 (2012). 22517619

[b45] Forde N. *et al.* Effects of Low Progesterone on the Endometrial Transcriptome in Cattle. Biology of Reproduction 87, 10.1095/biolreprod.112.103424 (2012). 23018184

[b46] Samborski A., Graf A., Krebs S., Kessler B. & Bauersachs S. Deep sequencing of the porcine endometrial transcriptome on day 14 of pregnancy. Biology of reproduction 88, 84 (2013). 23426436 10.1095/biolreprod.113.107870

[b47] Samborski A. *et al.* Transcriptome Changes in the Porcine Endometrium During the Pre-attachment Phase. Biology of reproduction. 89, 10.1095/biolreprod.113.112177 (2013). 24174570

[b48] Garg R. *et al.* Gene discovery and tissue-specific transcriptome analysis in chickpea with massively parallel pyrosequencing and web resource development. Plant Physiol. 156, 1661–1678 (2011). 21653784 10.1104/pp.111.178616 PMC3149962

[b49] Cánovas A., Rincon G., Islas-Trejo A., Wickramasinghe S. & Medrano J. F. SNP discovery in the bovine milk transcriptome using RNA-Seq technology. Mamm. Genome 21, 592–598 (2010). 21057797 10.1007/s00335-010-9297-z PMC3002166

[b50] Tóth G., Gáspári Z. & Jurka J. Microsatellites in different eukaryotic genomes: survey and analysis. Genome Res. 10, 967–981 (2000). 10899146 10.1101/gr.10.7.967 PMC310925

[b51] Li Y. C., Korol A. B., Fahima T., Beiles A. & Nevo E. Microsatellites: genomic distribution, putative functions and mutational mechanisms: a review. Mol. Ecol. 11, 2453–2465 (2002). 12453231 10.1046/j.1365-294x.2002.01643.x

[b52] Yagel S., Parhar R. S., Jeffrey J. J. & Lala P. K. Normal nonmetastatic human trophoblast cells share *in vitro* invasive properties of malignant cells. Journal of cellular physiology 136, 455–462 (1988). 3170642 10.1002/jcp.1041360309

[b53] Aplin J. D. Implantation, trophoblast differentiation and hæmochorial placentation: mechanistic evidence *in vivo* and *in vitro*. J Cell Sci 99, 681–692 (1991). 1769999 10.1242/jcs.99.4.681

[b54] Birkedal-Hansen H. *et al.* Matrix metalloproteinases: a review. Critical Reviews in Oral Biology & Medicine 4, 197–250 (1993). 8435466 10.1177/10454411930040020401

[b55] Harris L. K. *et al.* Trophoblast-and vascular smooth muscle cell-derived MMP-12 mediates elastolysis during uterine spiral artery remodeling. The American journal of pathology 177, 2103–2115 (2010). 20802175 10.2353/ajpath.2010.100182 PMC2947303

[b56] Brinckerhoff C. E. & Matrisian L. M. Timeline - Matrix metalloproteinases: a tail of a frog that became a prince. Nature Reviews Molecular Cell Biology 3, 207–214, 10.1038/nrm763 (2002). 11994741

[b57] Pardo A. & Selman M. MMP-1: the elder of the family. The international journal of biochemistry & cell biology 37, 283–288 (2005). 15474975 10.1016/j.biocel.2004.06.017

[b58] Bertini I. *et al.* Structural Basis for Matrix Metalloproteinase 1-Catalyzed Collagenolysis. Journal of the American Chemical Society 134, 2100–2110, 10.1021/ja208338j (2012). 22239621 PMC3298817

[b59] Emonard H., Christiane Y., Smet M., Grimaud J. & Foidart J.-M. Type IV and interstitial collagenolytic activities in normal and malignant trophoblast cells are specifically regulated by the extracellular matrix. Invasion & metastasis 10, 170–177 (1989). 2159447

[b60] Huppertz B., Kertschanska S., Demir A. Y., Frank H. G. & Kaufmann P. Immunohistochemistry of matrix metalloproteinases (MMP), their substrates, and their inhibitors (TIMP) during trophoblast invasion in the human placenta. Cell and Tissue Research 291, 133–148 (1998). 9394051 10.1007/s004410050987

[b61] Tatsumi K. *et al.* Expression of calcium binding protein D-9k messenger RNA in the mouse uterine endometrium during implantation. Mol. Human Reprod. 5, 153–161 (1999). 10.1093/molehr/5.2.153 10065871

[b62] Warembourg M., Perret C. & Thomasset M. Analysis and in situ detection of cholecalcin messenger RNA (9000 Mr CaBP) in the uterus of the pregnant rat. Cell and tissue research 247, 51–57 (1987). 3829120 10.1007/BF00216546

[b63] Krisinger J., Jeung E.-B., Simmen R. & Leung P. Porcine calbindin-D9k gene: expression in endometrium, myometrium, and placenta in the absence of a functional estrogen response element in intron A. Biology of reproduction 52, 115–123 (1995). 7711170 10.1095/biolreprod52.1.115

[b64] Choi Y., Seo H., Kim M. & Ka H. Dynamic expression of calcium-regulatory molecules, TRPV6 and S100G, in the uterine endometrium during pregnancy in pigs. Biology of reproduction 81, 1122–1130 (2009). 19641180 10.1095/biolreprod.109.076703

[b65] Nie G.-Y., Wang Y. L. J., Minoura H., Findlay J. K. & Salamonsen L. A. Complex regulation of calcium-binding protein D9k (Calbindin-D9k) in the mouse uterus during early pregnancy and at the site of embryo implantation. Biology of reproduction 62, 27–36 (2000). 10611064 10.1095/biolreprod62.1.27

[b66] Choi K. C. & Jeung E. B. Molecular mechanism of regulation of the calcium‐binding protein calbindin‐D9k, and its physiological role (s) in mammals: a review of current research. J. Cell. Mol. Med. 12, 409–420 (2008). 18182065 10.1111/j.1582-4934.2007.00209.x PMC3822532

[b67] Yun S. M. *et al.* Dominant expression of porcine Calbindin‐D9k in the uterus during a luteal phase. Mol. Reprod. Dev. 67, 251–256 (2004). 14735485 10.1002/mrd.20019

[b68] Supuran C. T., Fiore A. D. & Simone G. D. Carbonic anhydrase inhibitors as emerging drugs for the treatment of obesity. Expert Opinion on Emerging Drugs, 13, 383–392 (2008). 18537527 10.1517/14728214.13.2.383

[b69] Tufts B., Esbaugh A. & Lund S. Comparative physiology and molecular evolution of carbonic anhydrase in the erythrocytes of early vertebrates. Comparative Biochemistry and Physiology Part A: Molecular & Integrative Physiology 136, 259–269 (2003). 10.1016/s1095-6433(03)00159-4 14511745

[b70] Sowden J., Leigh S., Talbot I., Delhanty J. & Edwards Y. Expression from the proximal promoter of the carbonic anhydrase 1 gene as a marker for differentiation in colon epithelia. Differentiation 53, 67–74 (1993). 8359594 10.1111/j.1432-0436.1993.tb00647.x

[b71] Wang R.-J. *et al.* Down-regulated MYH11 expression correlates with poor prognosis in stage II and III colorectal cancer. Asian Pac J Cancer Prev 15, 7223–7228 (2014). 25227818 10.7314/apjcp.2014.15.17.7223

[b72] Matsuoka R. *et al.* Human smooth muscle myosin heavy chain gene mapped to chromosomal region 16q12. American journal of medical genetics 46, 61–67 (1993). 7684189 10.1002/ajmg.1320460110

[b73] Loikkanen I. *et al.* Myosin VI is a modulator of androgen-dependent gene expression. Oncol. Rep. 22, 991 (2009). 19787211 10.3892/or_00000526

[b74] Cui W.-j. *et al.* Myosin light chain kinase is responsible for high proliferative ability of breast cancer cells via anti-apoptosis involving p38 pathway. Acta Pharmacologica Sinica 31, 725–732 (2010). 20453870 10.1038/aps.2010.56 PMC4002978

[b75] Pessina P. *et al.* Skeletal muscle of gastric cancer patients expresses genes involved in muscle regeneration. Oncol. Rep. 24, 741–745 (2010). 20664982 10.3892/or_00000916

[b76] Yang L. *et al.* Role of MYH Polymorphisms in Sporadic Colorectal Cancer in China: A Case-control, Population-based Study. Asian Pacific Journal of Cancer Prevention 14, 6403–6409 (2013). 24377541 10.7314/apjcp.2013.14.11.6403

[b77] Seitz S. *et al.* Genetic background of different cancer cell lines influences the gene set involved in chromosome 8 mediated breast tumor suppression. Genes Chromosomes Cancer 45, 612–627 (2006). 16552773 10.1002/gcc.20325

[b78] Lu Y. *et al.* Cross-species comparison of orthologous gene expression in human bladder cancer and carcinogen-induced rodent models. American journal of translational research 3, 8 (2011). 21139803 PMC2981423

[b79] Collette T. *et al.* Evidence for an increased release of proteolytic activity by the eutopic endometrial tissue in women with endometriosis and for involvement of matrix metalloproteinase‐9. Human Reproduction 19, 1257–1264 (2004). 15105396 10.1093/humrep/deh290

[b80] Cosín R. *et al.* Influence of peritoneal fluid on the expression of angiogenic and proteolytic factors in cultures of endometrial cells from women with endometriosis. Human reproduction 25, 398–405 (2010). 19945964 10.1093/humrep/dep419

[b81] Béliard A., Noël A. & Foidart J.-M. Reduction of apoptosis and proliferation in endometriosis. Fertility and sterility 82, 80–85 (2004). 15236993 10.1016/j.fertnstert.2003.11.048

[b82] Kokawa K., Shikone T. & Nakano R. Apoptosis in the human uterine endometrium during the menstrual cycle. The Journal of Clinical Endocrinology & Metabolism 81, 4144–4147 (1996). 8923873 10.1210/jcem.81.11.8923873

[b83] Li M.-Q. *et al.* CXCL8 enhances proliferation and growth and reduces apoptosis in endometrial stromal cells in an autocrine manner via a CXCR1-triggered PTEN/AKT signal pathway. Human reproduction 27, 2107–2116 (2012). 22563025 10.1093/humrep/des132

[b84] Szymanowski K. Apoptosis pattern in human endometrium in women with pelvic endometriosis. European Journal of Obstetrics & Gynecology and Reproductive Biology 132, 107–110 (2007). 16698169 10.1016/j.ejogrb.2006.04.008

[b85] Suzuki N. *et al.* A cytoplasmic protein, bystin, interacts with trophinin, tastin, and cytokeratin and may be involved in trophinin-mediated cell adhesion between trophoblast and endometrial epithelial cells. Proceedings of the National Academy of Sciences 95, 5027–5032 (1998). 10.1073/pnas.95.9.5027 PMC20207 9560222

[b86] Pandey A., Singh N., Gupta S., Rana J. & Gupta N. Relative expression of cell growth regulatory genes insulin-like growth factors (IGF-1 and IGF-2) and their receptors (IGF-1R and IGF-2R) in somatic cell nuclear transferred (SCNT) and *in vitro* fertilized (IVF) pre-implantation buffalo embryos. Cell Biol. Int. 33, 555–564 (2009). 19281854 10.1016/j.cellbi.2009.02.013

[b87] Rutanen E.-M. Insulin-like growth factors in endometrial function. Gynecological Endocrinology 12, 399–406 (1998). 10065165 10.3109/09513599809012842

[b88] Soufla G., Sifakis S. & Spandidos D. A. FGF2 transcript levels are positively correlated with EGF and IGF-1 in the malignant endometrium. Cancer Lett. 259, 146–155 (2008). 18006148 10.1016/j.canlet.2007.10.002

[b89] Giudice L. *et al.* Physiology: Insulin-like growth factor (IGF), IGF binding protein (IGFBP), and IGF receptor gene expression and IGFBP synthesis in human uterine leiomyomata. Human Reproduction 8, 1796–1806 (1993). 7507128 10.1093/oxfordjournals.humrep.a137937

[b90] Carmeliet P. & Jain R. K. Angiogenesis in cancer and other diseases. Nature 407, 249–257 (2000). 11001068 10.1038/35025220

[b91] Kayisli O. G., Kayisli U. A., Luleci G. & Arici A. *In vivo* and *in vitro* regulation of Akt activation in human endometrial cells is estrogen dependent. Biology of reproduction 71, 714–721 (2004). 15115729 10.1095/biolreprod.104.027235

[b92] Tong W. & Pollard J. W. Progesterone inhibits estrogen-induced cyclin D1 and cdk4 nuclear translocation, cyclin E-and cyclin A-cdk2 kinase activation, and cell proliferation in uterine epithelial cells in mice. Mol. Cell. Biol. 19, 2251–2264 (1999). 10022912 10.1128/mcb.19.3.2251 PMC84018

[b93] Kuokkanen S. *et al.* Genomic profiling of microRNAs and messenger RNAs reveals hormonal regulation in microRNA expression in human endometrium. Biology of reproduction 82, 791–801 (2010). 19864316 10.1095/biolreprod.109.081059 PMC2842492

[b94] Bartosik D., Jacobs S. & Kelly L. Endometrial tissue in peritoneal fluid. Fertility and sterility 46, 796–800 (1986). 3780999 10.1016/s0015-0282(16)49813-4

[b95] Laherty C. *et al.* Characterization of mouse thrombospondin 2 sequence and expression during cell growth and development. J. Biol. Chem. 267, 3274–3281 (1992). 1371115

[b96] Haouzi D. *et al.* Transcriptome analysis reveals dialogues between human trophectoderm and endometrial cells during the implantation period. Human reproduction 26, 1440–1449 (2011). 21427117 10.1093/humrep/der075

[b97] Zhang R.-J. *et al.* Functional expression of large-conductance calcium-activated potassium channels in human endometrium: a novel mechanism involved in endometrial receptivity and embryo implantation. The Journal of Clinical Endocrinology & Metabolism 97, 543–553 (2011). 22170720 10.1210/jc.2011-2108

[b98] Liu X.-M. *et al.* Down-regulation of S100A11, a calcium-binding protein, in human endometrium may cause reproductive failure. The Journal of Clinical Endocrinology & Metabolism 97, 3672–3683 (2012). 22869607 10.1210/jc.2012-2075 PMC3462935

[b99] Siebert C. *et al.* Effect of physical exercise on changes in activities of creatine kinase, cytochrome c oxidase and ATP levels caused by ovariectomy. Metab. Brain Dis. 29, 825–835, doi: 10.1007/s11011-014-9564-x (2014). 24810635 10.1007/s11011-014-9564-x

[b100] Burghardt R. C. *et al.* Integrins and extracellular matrix proteins at the maternal-fetal interface in domestic animals. Cells, tissues, organs 172, 202–217 (2001). 12476049 10.1159/000066969

[b101] Kim M. *et al.* Activated leukocyte cell adhesion molecule: expression in the uterine endometrium during the estrous cycle and pregnancy in pigs. Asian-Aust J Anim Sci 24, 919–928 (2011).

[b102] Lay V., Yap J., Sonderegger S. & Dimitriadis E. Interleukin 11 regulates endometrial cancer cell adhesion and migration via STAT3. International journal of oncology 41, 759–764 (2012). 22614117 10.3892/ijo.2012.1486

[b103] Bosse T. *et al.* L1 cell adhesion molecule is a strong predictor for distant recurrence and overall survival in early stage endometrial cancer: Pooled PORTEC trial results. European Journal of Cancer 50, 2602–2610 (2014). 25126672 10.1016/j.ejca.2014.07.014

[b104] Willoughby D. & Cooper D. M. Organization and Ca2+ regulation of adenylyl cyclases in cAMP microdomains. Physiol. Rev. 87, 965–1010 (2007). 17615394 10.1152/physrev.00049.2006

[b105] Wayman G. A. *et al.* Synergistic activation of the type I adenylyl cyclase by Ca2+ and Gs-coupled receptors *in vivo*. J. Biol. Chem. 269, 25400–25405 (1994). 7929237

[b106] Nielsen M. D., Chan G. C., Poser S. W. & Storm D. R. Differential regulation of type I and type VIII Ca2+-stimulated adenylyl cyclases by Gi-coupled receptors *in vivo*. J. Biol. Chem. 271, 33308–33316 (1996). 8969190 10.1074/jbc.271.52.33308

[b107] de Mooij-van Malsen A. J. *et al.* Interspecies Trait Genetics Reveals Association of Adcy8 with Mouse Avoidance Behavior and a Human Mood Disorder. Biological psychiatry 66, 1123–1130 (2009). 10.1016/j.biopsych.2009.06.016 19691954

[b108] Zimmermann D. R. & Ruoslahti E. Multiple domains of the large fibroblast proteoglycan, versican. The EMBO journal 8, 2975 (1989). 2583089 10.1002/j.1460-2075.1989.tb08447.x PMC401368

[b109] Burns T. A. *et al.* Imbalanced Expression of Vcan mRNA Splice Form Proteins Alters Heart Morphology and Cellular Protein Profiles. PloS one 9, e89133 (2014). 24586547 10.1371/journal.pone.0089133 PMC3930639

[b110] Rahmani M. *et al.* Versican: signaling to transcriptional control pathways This paper is one of a selection of papers published in this Special Issue, entitled Young Investigator’s Forum. Canadian journal of physiology and pharmacology 84, 77–92 (2006). 16845893 10.1139/y05-154

[b111] Mukhopadhyay A. *et al.* Erosive vitreoretinopathy and wagner disease are caused by intronic mutations in CSPG2/Versican that result in an imbalance of splice variants. Investigative ophthalmology & visual science 47, 3565–3572 (2006). 16877430 10.1167/iovs.06-0141

[b112] Gillan L. *et al.* Periostin secreted by epithelial ovarian carcinoma is a ligand for αVβ3 and αVβ5 integrins and promotes cell motility. Cancer Res. 62, 5358–5364 (2002). 12235007

[b113] Yan W. & Shao R. Transduction of a mesenchyme-specific gene periostin into 293T cells induces cell invasive activity through epithelial-mesenchymal transformation. J. Biol. Chem. 281, 19700–19708 (2006). 16702213 10.1074/jbc.M601856200

[b114] Edgell C.-J. S., BaSalamah M. A. & Marr H. S. Testican-1: a differentially expressed proteoglycan with protease inhibiting activities. Int. Rev. Cytol. 236, 101–122 (2004). 15261737 10.1016/S0074-7696(04)36003-1

[b115] Hausser H.-J., Decking R. & Brenner R. E. Testican-1, an inhibitor of pro-MMP-2 activation, is expressed in cartilage. Osteoarthritis and cartilage 12, 870–877 (2004). 15501402 10.1016/j.joca.2004.07.008

[b116] Röll S., Seul J., Paulsson M. & Hartmann U. Testican-1 is dispensable for mouse development. Matrix biology 25, 373–381 (2006). 16806869 10.1016/j.matbio.2006.05.004

[b117] Dhamija R., Graham J. M. Jr, Smaoui N., Thorland E. & Kirmani S. Novel de novo SPOCK1 mutation in a proband with developmental delay, microcephaly and agenesis of corpus callosum. European journal of medical genetics 57, 181–184 (2014) 24583203 10.1016/j.ejmg.2014.02.009

[b118] Miao L. Y. *et al.* SPOCK1 is a novel transforming growth factor-beta target gene that regulates lung cancer cell epithelial-mesenchymal transition. Biochem. Biophys. Res. Commun. 440, 792–797, 10.1016/j.bbrc.2013.10.024 (2013). 24134845

[b119] Li Y. *et al.* SPOCK1 is regulated by CHD1L and blocks apoptosis and promotes HCC cell invasiveness and metastasis in mice. Gastroenterology 144, 179–191, e174 (2013). 23022495 10.1053/j.gastro.2012.09.042

[b120] Wlazlinski A. *et al.* Downregulation of several fibulin genes in prostate cancer. The prostate 67, 1770–1780 (2007). 17929269 10.1002/pros.20667

[b121] Chen H., Herndon M. E. & Lawler J. The cell biology of thrombospondin-1. Matrix Biology 19, 597–614 (2000). 11102749 10.1016/s0945-053x(00)00107-4

[b122] Bonazzi V. F. *et al.* Cross-platform array screening identifies COL1A2, THBS1, TNFRSF10D and UCHL1 as genes frequently silenced by methylation in melanoma. PloS one 6, e26121 (2011). 22028813 10.1371/journal.pone.0026121 PMC3197591

[b123] Isenberg J. S., Martin-Manso G., Maxhimer J. B. & Roberts D. D. Regulation of nitric oxide signalling by thrombospondin 1: implications for anti-angiogenic therapies. Nature Reviews Cancer 9, 182–194 (2009). 19194382 10.1038/nrc2561 PMC2796182

[b124] Alvarez M. C., Ladeira M. S. P., Scaletsky I. C. A., Pedrazzoli J. Jr & Ribeiro M. L. Methylation Pattern of THBS1, GATA-4, and HIC1 in Pediatric and Adult Patients Infected with Helicobacter pylori. Dig. Dis. Sci. 58, 2850–2857 (2013). 23765259 10.1007/s10620-013-2742-6

[b125] Oguri M. *et al.* Association of polymorphisms of THBS2 and HSPA8 with hypertension in Japanese individuals with chronic kidney disease. Molecular medicine reports 2, 205–211 (2009). 21475814 10.3892/mmr_00000085

[b126] Schroen B. *et al.* Thrombospondin-2 Is Essential for Myocardial Matrix Integrity Increased Expression Identifies Failure-Prone Cardiac Hypertrophy. Circul. Res. 95, 515–522 (2004). 10.1161/01.RES.0000141019.20332.3e 15284191

[b127] Daniel C., Amann K., Hohenstein B., Bornstein P. & Hugo C. Thrombospondin 2 functions as an endogenous regulator of angiogenesis and inflammation in experimental glomerulonephritis in mice. Journal of the American Society of Nephrology 18, 788–798 (2007). 17287428 10.1681/ASN.2006080873

[b128] Igwebuike U. A review of uterine structural modifications that influence conceptus implantation and development in sheep and goats. Animal reproduction science 112, 1–7 (2009). 19162416 10.1016/j.anireprosci.2008.12.010

[b129] Yates S. A. *et al.* De novo assembly of red clover transcriptome based on RNA-Seq data provides insight into drought response, gene discovery and marker identification. BMC Genomics 15, 453 (2014). 24912738 10.1186/1471-2164-15-453 PMC4144119

[b130] Ashburner M. *et al.* Gene Ontology: tool for the unification of biology. Nat. Genet. 25, 25–29 (2000). 10802651 10.1038/75556 PMC3037419

[b131] Consortium G. O. The Gene Ontology (GO) database and informatics resource. Nucleic Acids Res. 32, D258–D261 (2004). 14681407 10.1093/nar/gkh036 PMC308770

[b132] Ji Z. *et al.* Identification of novel and differentially expressed microRNAs of dairy goat mammary gland tissues using Solexa sequencing and bioinformatics. PloS one 7, e49463 (2012). 23166677 10.1371/journal.pone.0049463 PMC3498112

[b133] Kanehisa M. *et al.* KEGG for linking genomes to life and the environment. Nucleic Acids Res. 36, D480–D484 (2008). 18077471 10.1093/nar/gkm882 PMC2238879

[b134] Brousseau L. *et al.* High-throughput transcriptome sequencing and preliminary functional analysis in four Neotropical tree species. BMC Genomics 15, 238 (2014). 24673733 10.1186/1471-2164-15-238 PMC3986928

[b135] Fournier-Level A. *et al.* A map of local adaptation in Arabidopsis thaliana. Science 334, 86–89 (2011). 21980109 10.1126/science.1209271

[b136] Hancock A. M. *et al.* Adaptation to climate across the Arabidopsis thaliana genome. Science 334, 83–86 (2011). 21980108 10.1126/science.1209244

[b137] Siol M., Wright S. I. & Barrett S. C. The population genomics of plant adaptation. New Phytol. 188, 313–332 (2010). 20696011 10.1111/j.1469-8137.2010.03401.x

[b138] You F. M. *et al.* BatchPrimer3: a high throughput web application for PCR and sequencing primer design. BMC Bioinformatics 9, 253 (2008). 18510760 10.1186/1471-2105-9-253 PMC2438325

