# Supplementary material for: Characterization of the Transcriptional Complexity of the Receptive and Pre-receptive Endometria of Dairy Goats
Source: Sci Rep. 2015 Sep 16;5:14244. doi: 10.1038/srep14244 (PMC4571617; doi:10.1038/srep14244)
Supplement: Supporting Information [file srep14244-s10.pdf]

# Characterization of the Transcriptional Complexity of the Receptive and Pre-receptive Endometria of Dairy Goats

**Lei Zhang, Xiao-Peng An, Yu-Xuan Song*, Xiao-Rui, Liu, Ming-Zhe Fu, Peng Han, Jia-Yin Peng, Jing-Xing Hou, Zhan-Qin Zhou, Bin-Yun Cao**

## Supporting Information:

Table S1 | The result of unigene annotation. (XLS)

Table S2 | List of the putative SNPs in PE of dairy goat. (XLS)

Table S3 | List of the putative SNPs in RE of dairy goat. (XLS)

Table S4 | List of the putative SSRs in dairy goat. (XLS)

Table S5 | The full list of DEGs with FDR-value was less than 0.001. (XLS)

Table S6 | Gene Ontology (GO) assignment of the DEGs for the molecular functions category. (XLS)

Table S7 | Gene Ontology (GO) assignment of the DEGs for the cellular compartment category. (XLS)

Table S8 | Gene Ontology (GO) assignment of the DEGs for the biological processes category. (XLS)

Table S9 | List of KEGG pathway categories for DEGs between PE and RE. (XLS)
